# Highly Pathogenic Avian Influenza A(H5N8) Virus Clade 2.3.4.4b, Western Siberia, Russia, 2020

**DOI:** 10.3201/eid2708.204969

**Published:** 2021-08

**Authors:** Ivan Sobolev, Kirill Sharshov, Nikita Dubovitskiy, Olga Kurskaya, Alexander Alekseev, Sergey Leonov, Yuriy Yushkov, Victor Irza, Andrey Komissarov, Artem Fadeev, Daria Danilenko, Junki Mine, Ryota Tsunekuni, Yuko Uchida, Takehiko Saito, Alexander Shestopalov

**Affiliations:** Federal Research Center of Fundamental and Translational Medicine, Novosibirsk, Russia (I. Sobolev, K. Sharshov, N. Dubovitskiy, O. Kurskaya, A. Alekseev, A. Shestopalov);; Siberian Federal Scientific Centre of Agro- BioTechnologies, Krasnoobsk, Russia (S. Leonov, Y. Yushkov);; Federal Governmental State-Financed Institution Federal Centre for Animal Health, Vladimir, Russia (V. Irza);; Smorodintsev Research Institute of Influenza, St. Petersburg, Russia (A. Komissarov, A. Fadeev, D. Danilenko);; Division of Transboundary Animal Disease, National Institute of Animal Health, Tsukuba, Japan (J. Mine, R. Tsunekuni, Y. Uchida, T. Saito)

**Keywords:** influenza, H5N8, reassortment, avian influenza virus, clade 2.3.4.4b, Western Siberia, Russia, HPAIV, LPAIV, viruses, respiratory infections, zoonoses

## Abstract

Two variants of highly pathogenic avian influenza A(H5N8) virus were detected in dead poultry in Western Siberia, Russia, during August and September 2020. One variant was represented by viruses of clade 2.3.4.4b and the other by a novel reassortant between clade 2.3.4.4b and Eurasian low pathogenicity avian influenza viruses circulating in wild birds.

In 1996, the highly pathogenic avian influenza (HPAI) A(H5N1) virus subtype of the A/goose/Guangdong/1/1996 lineage was detected in domestic geese in China (*1*). Since 2014, H5Nx HPAI viruses belonging to clade 2.3.4.4 of A/goose/Guangdong/1/1996 lineage have spread internationally, posing a threat to the health of poultry and wild birds. Viruses of clade 2.3.4.4b have been detected in China (2013) and South Korea (2014); in 2016, reassortant strains between 2.3.4.4b and the Eurasian low pathogenicity avian influenza (LPAI) virus, for polymerase basic protein 2 (PB2), polymerase basic protein 1 (PB1), polymerase acidic gene (PA), nucleoprotein (NP), and matrix gene (M) segments, were reported in China (Qinghai Lake) and Russia (Uvs–Nuur Lake) (*2*). Thereafter, 2.3.4.4b viruses and their reassortant strains have spread worldwide and have been identified in poultry and wild birds in multiple countries (*3*).

In January and February 2020, a novel HPAI H5N8 clade 2.3.4.4b virus was detected in Germany. This virus shares 6 gene segments with the HPAI H5N8 virus in Eurasia, Asia, and Africa and 2 gene segments with LPAI virus A(H3N8), which has recently been detected in wild birds of Russia (*4*). HPAI virus strains closely related to isolates from Germany have also been identified in other countries of Europe, according to GISAID (https://www.gisaid.org). In October 2020, HPAI virus related to the variant from Germany has also been isolated in Japan (*5*) and South Korea (*6*).

Other variants of HPAI H5Nx virus were detected in the fall of 2020. Viruses of genetic group B of clade 2.3.4.4 and subtypes H5N8, H5N5, and H5N1 were found in Russia, Kazakhstan, and a number of countries in Europe (*3*,*7*,*8*). These viruses are genetically related to strains isolated in Egypt during 2017–2019 (*7*) and in Iraq in May 2020 (*8*).

The previous cases of H5 HPAI virus in Russia occurred at the end of 2018. In 2019 and the first half of 2020 H5Nx viruses had not been detected in Russia. In August and September 2020, we collected 58 samples from dead domestic birds on private rural farms in Western Siberia. We characterized 7 strains by using complete genome sequencing, phylogenetic analysis, and intravenous pathogenicity index testing. We identified all 7 strains as HPAI viruses on the basis of the amino acid sequence of the hemagglutinin (HA) proteolytic cleavage site (PLREKRRKR|G) and intravenous pathogenicity index values of 2.92–2.93 in chickens (Table).

**Table:** Highly pathogenic avian influenza A viruses subtype H5N8 isolated from birds, Novosibirsk, Western Siberia, Russia, 2020*

Group	Isolate ID	Site	Collection date	IVPI value
1	A/goose/Russia_Novosibirsk region/1-12/2020	Intestine	2020 Sep 15	2.92
1	A/goose/Russia_Omsk region/55-1/2020	Intestine	2020 Aug 29	2.92
1	A/chicken/Russia_Novosibirsk region/1910-1/2020	Liver	2020 Sep 22	2.92
1	A/chicken/Russia_Novosibirsk region/1910-2/2020	Intestine	2020 Sep 22	2.92
2	A/chicken/Russia_Novosibirsk region/3-1/2020	Intestine	2020 Sep 20	2.93
2	A/chicken/Russia_Novosibirsk region/3-15/2020	Intestine	2020 Sep 20	2.93
2	A/chicken/Russia_Novosibirsk region/3-29/2020	Brain	2020 Sep 20	2.93

*ID, identification; IVPI, intravenous pathogenicity index.

We divided the isolated strains into 2 groups according to the sequences of the genome segments. Group 1 consists of 4 strains, whereas group 2 consists of 3 strains (Table). By using BLAST analysis (https://blast.ncbi.nlm.nih.gov/Blast.cgi), we found all 8 genome segments of group 1 and the 3 genome segments (HA, M, and NS) of group 2 to be closely related (99.01%–100% nucleotide identity) to the genome segments of HPAI clade 2.3.4.4b virus strains isolated in Russia, Kazakhstan, and Europe in the summer and fall of 2020. We found the genome segments of neuraminidase, PB2, PB1, PA, and NP in group 2 to be related (98.38%–99.06% nucleotide identity) to different LPAI viruses from Eurasia.

Phylogenetic analysis showed that the whole genome of group 1 and HA, M, and nonstructural gene genome segments of group 2 clustered with HPAI H5N8 clade 2.3.4.4b virus. They were also related to H5N8 viruses from Egypt (2019) and Iraq (May 2020) but were not related to the H5N8 variants from Germany in early 2020 (Figure; Appendix 1 Figures 1–7). The neuraminidase, PB2, PB1, PA, and NP segments of group 2 viruses clustered with LPAI viruses identified in Eurasia. Consequently, group 2 strains are reassortant strains between Egyptian-like HPAI and LPAI viruses from Eurasia (Appendix 1 Figure 8). Of note, PB2, PA, and NP segments of group 2 isolates clustered on phylogenetic trees (nucleotide identity of 97.32%–97.45% for PB2, 98.98%–99.02% for PA, and 98.86%–99.00% for NP) with the HPAI H5N1 reassortants isolated in the fall of 2020 in the Netherlands (*8*). PB1 segments showed a lower level of identity (96.21%–96.26%).

**Figure:**
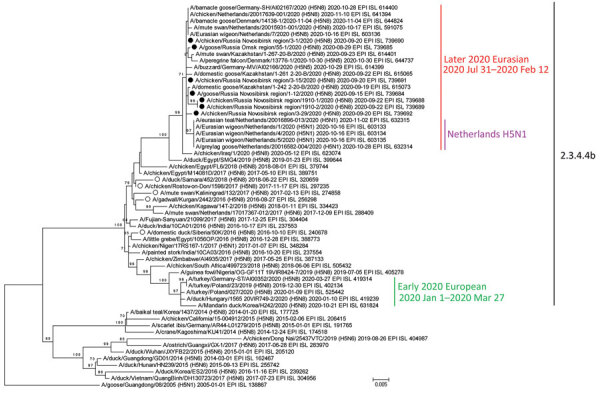
Maximum-likelihood phylogenetic tree of the hemagglutinin segment of HPAI subtype H5N8 virus isolated from birds, Novosibirsk, Western Siberia, Russia, 2020, and reference segments from GISAID (http://www.gisaid.org). Filled circles indicate HPAI H5N8 virus strains from Russia isolated in 2020; open circles indicate strains from Russia isolated during 2016–2018. Virus identification number, date of identification, and GISAID accession number are provided for all sequences. HPAI, highly pathogenic avian influenza.

On the basis of our phylogenetic data, chronology of virus isolations, general birds’ flyways, and previously described patterns of HPAI viruses spreading from Siberia during 2005–2006, 2014, and 2016–2017 (*3*,*9*,*10*), we suggest that new H5N8 viral strain from Eurasia in late 2020 possibly descended from the H5N8 virus circulating in Egypt during 2017–2019 and then disseminated through Iraq into Western Siberia and North Kazakhstan during the spring migration. Egyptian-like HPAI H5N8 virus possibly reached breeding and staging areas in Siberia in early 2020, spread in wild bird populations, and reassorted with LPAI viruses. During fall migration, standard Egyptian-like HPAI H5N8 virus and novel reassortant strains spread to the European part of Eurasia, leading to a reassortment event, which has been detected in Netherlands. However, further studies of 2020–2021 European H5Nx viruses are needed to verify this hypothesis.

Appendix 1 Materials and methods for study of highly pathogenic avian influenza A(H5N8) virus clade 2.3.4.4b, Western Siberia, Russia.

Appendix 2 GISAID authors providing sequence information in support of study of highly pathogenic avian influenza A(H5N8) virus clade 2.3.4.4b, Western Siberia, Russia, 2020.
