# Supplementary material for: Highly Pathogenic Avian Influenza A(H5N8) Virus Clade 2.3.4.4b, Western Siberia, Russia, 2020
Source: Emerg Infect Dis. 2021 Aug;27(8):2224–7. doi: 10.3201/eid2708.204969 (PMC8314819; doi:10.3201/eid2708.204969)
Supplement: Appendix 1 — Materials and methods for study of highly pathogenic avian influenza A(H5N8) virus clade 2.3.4.4b, Western Siberia, Russia. [file 20-4969-Techapp-s1.pdf]

Article DOI: https://doi.org/10.3201/eid2708.204969

Highly Pathogenic Avian Influenza A(H5N8) Virus Clade 2.3.4.4b, Western Siberia, Russia, 2020

Appendix 1

Materials and Methods

Samples

We collected 58 samples from dead domestic birds (chickens and geese) from small, private, village farms in Western Siberia between August and September 2020. In 30 of these samples, influenza virus H5 subtype were detected using real-time polymerase chain reaction (AmpliSens Influenza virus A H5N1-FRT PCR kit, AmpliSens, Russia). A total of 15 isolates of H5 viruses were isolated from 10-day-old chicken embryonating eggs using chicken embryo inoculation. All viruses caused the death of chicken embryos within two days. Samples and isolates were demonstrated to be H5 positive using real-time PCR.

It should be noted that samples were collected from small, private, rural farms, which have several of the following features. First, they allow for free grazing of poultry; ducks and geese can visit water bodies, such as lakes, near the village. Second, small, private, rural farms lack any biosafety measures. Third, birds of different species often cohabit the farm, which ensures transmission of the virus to chickens from geese or ducks that visit the lake and interact with wild birds there.

Therefore, they are convenient points for HPAIVs surveillance. However, owing to the lack of control over the activities of such small farms, it is difficult to clearly understand the causes and parameters of viral transmission on such farms. We assume that the reason is complex; poultry on free grazing farms interact with wild birds, and the lack of biosafety measures and the residence of several species of birds in a limited area of the farm promotes the spread of viruses across populations and species.

The selection of seven isolates for the further study was based on the "one farm – one isolate" principle. Isolates with the same collection date (A/chicken/Russia_Novosibirsk region/3-1/2020, A/chicken/Russia_Novosibirsk region/3-15/2020 and A/chicken/Russia_Novosibirsk region/3-29/2020 – 20.09.2020; A/chicken/Russia_Novosibirsk region/1910-1/2020 and A/chicken/Russia_Novosibirsk region/1910-2/2020 – 22.09.2020) were collected from different farms but in one village.

Genome sequencing and phylogenetic analysis

Complete genome sequencing of 7 influenza H5N8 isolates was performed by nanopore sequencing technology (Oxford-Nanopore MinION). RNA from was extracted using QIAamp Viral RNA Mini Kit. Whole-genome amplification of Influenza A virus genome was performed using protocol by Zhou et al. (*1*) Primers were modified by adding ONT universal tags: 5′-TTTCTGTTGGTGCTGATATTGC-3′ and 5′-ACTTGCCTGTCGCTCTATCTTC-3′ for forward and reverse primers, respectively. 1D Ligation sequencing kit (SQK-LSK109) with PCR barcoding expansion (EXP-PBC096) was utilized for sequencing library preparation. MinION (Oxford Nanopore) (flow cell R9.4.1) was used for whole-genome sequencing. Fast5 files produced by minION were basecalled and demultiplexed using guppy v3.6.0. Reads were mapped onto the reference sequence using minimap2 v2.17 with default settings. SAMtools-mpileup v1.10 was used to produce consensus sequences.

Nucleotide sequences were deposited in the GISAID database under accession number EPI_ISL_739684-EPI_ISL_739692. Phylogenetic trees were reconstructed using sequences of H5N8 influenza viruses isolated worldwide since 2016 from BLAST (GISAID). Initial Maximum-likelihood phylogenies for each of the gene segments were generated with RAxML (*2*) using the general time-reversible nucleotide substitution model. Final dendrograms were generated and visualized with MEGA5 (*3*). Bootstrap support values were generated using 1,000 rapid bootstrap replicates.

Migration studies

We considered the map (*4*) of the generalized waterbirds' flyways (Wetlands International group) and performed large-scale analysis of movements of waterbirds in Southwest Siberian. Locations of virus isolations on a map for waterbird migration (Wader Flyways), according to the Wetlands International group suggests, that the most likely way of

Egyptian-like virus spread with waterbird spring migration might be along the Asian–East African or/and Mediterranean/Black Sea flyways. This was verified by large-scale analysis of ring recoveries from waterbirds in Eurasia (*4*).

Intravenous pathogenicity index (IVPI)

All animal experiments were approved by the Ethics Committee of the Federal Research Centre of Fundamental and Translational Medicine (Ethics Committee decision Nº36/1, 19.11.2020). For the determination of IVPI of 9 viruses, 0.1 ml of 1:10 dilution of infectious allantoic fluids were inoculated intravenously into ten 6-week-old specific pathogen free chickens. The IVPI was calculated according to the OIE standard protocol (available at: http://www.oie.int/international-standard-setting/terrestrial-code/). Isolates with an IVPI > 1.2 were determined to be HPAIV. The challenge study and all experiments with live viruses were conducted in a biosafety level 3 facility.

References

1. Zhou B, Donnelly ME, Scholes DT, St George K, Hatta M, Kawaoka Y, et al. Single-reaction genomic amplification accelerates sequencing and vaccine production for classical and swine origin human influenza a viruses. J Virol. 2009;83:10309–13. PubMed https://doi.org/10.1128/JVI.01109-09

2. Stamatakis A. RAxML version 8: a tool for phylogenetic analysis and post-analysis of large phylogenies. Bioinformatics. 2014;30:1312–3. PubMed https://doi.org/10.1093/bioinformatics/btu033

3. Tamura K, Peterson D, Peterson N, Stecher G, Nei M, Kumar S. MEGA5: molecular evolutionary genetics analysis using maximum likelihood, evolutionary distance, and maximum parsimony methods. Mol Biol Evol. 2011;28:2731–9. PubMed https://doi.org/10.1093/molbev/msr121

4. Veen J, Yurlov AK, Delany SN, Mihantiev AI, Selivanova MA, Boere GC. An atlas of movements of Southwest Siberian waterbirds. Wageningen (Netherlands): Wetlands International; 2005.

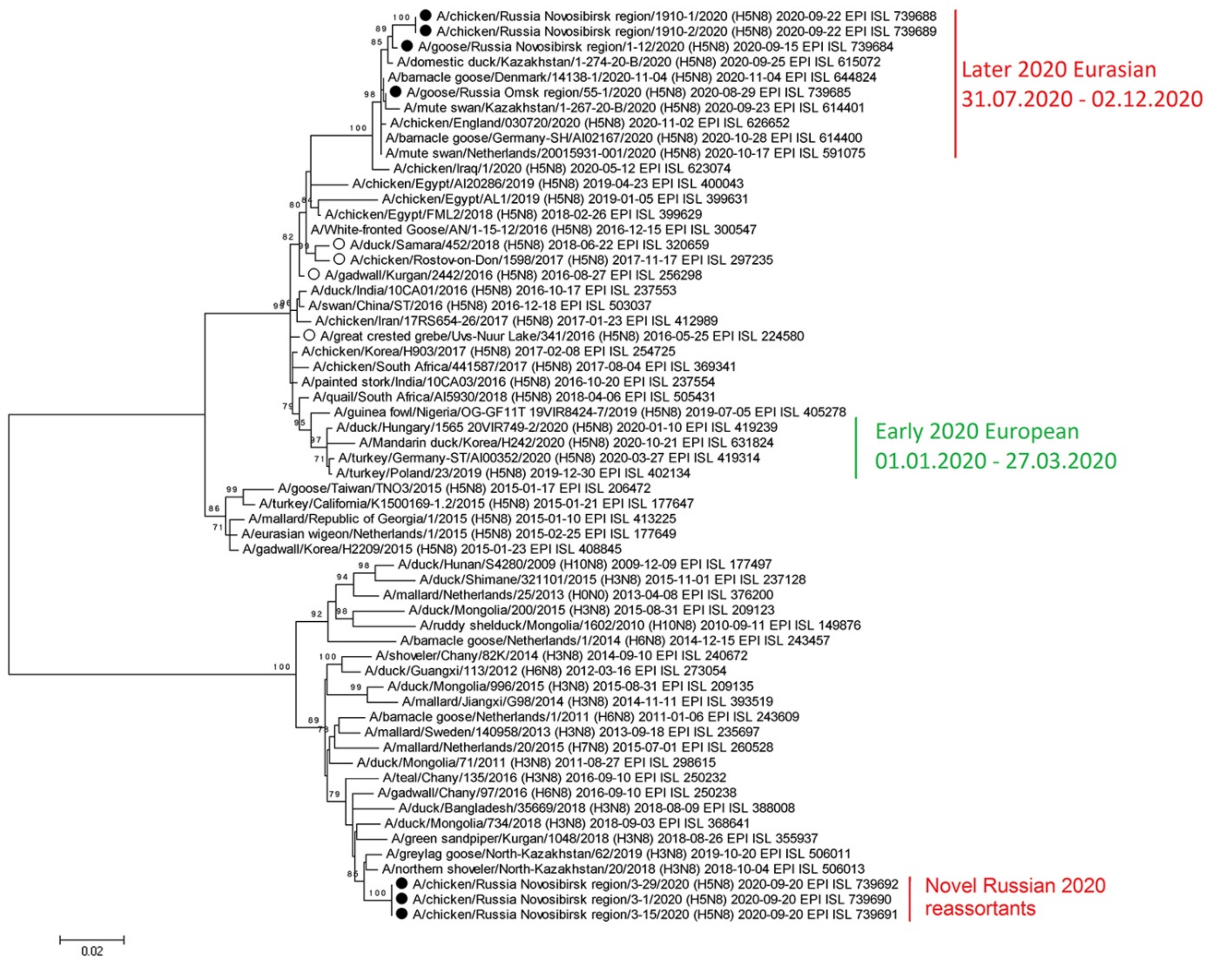

Appendix 1 Figure 1. Maximum-likelihood phylogenetic tree of the NA segment. Black circles - HPAIVs H5N8 from Russia isolated in 2020. White circles - Russian HPAIVs H5N8 isolated in 2016-2018.

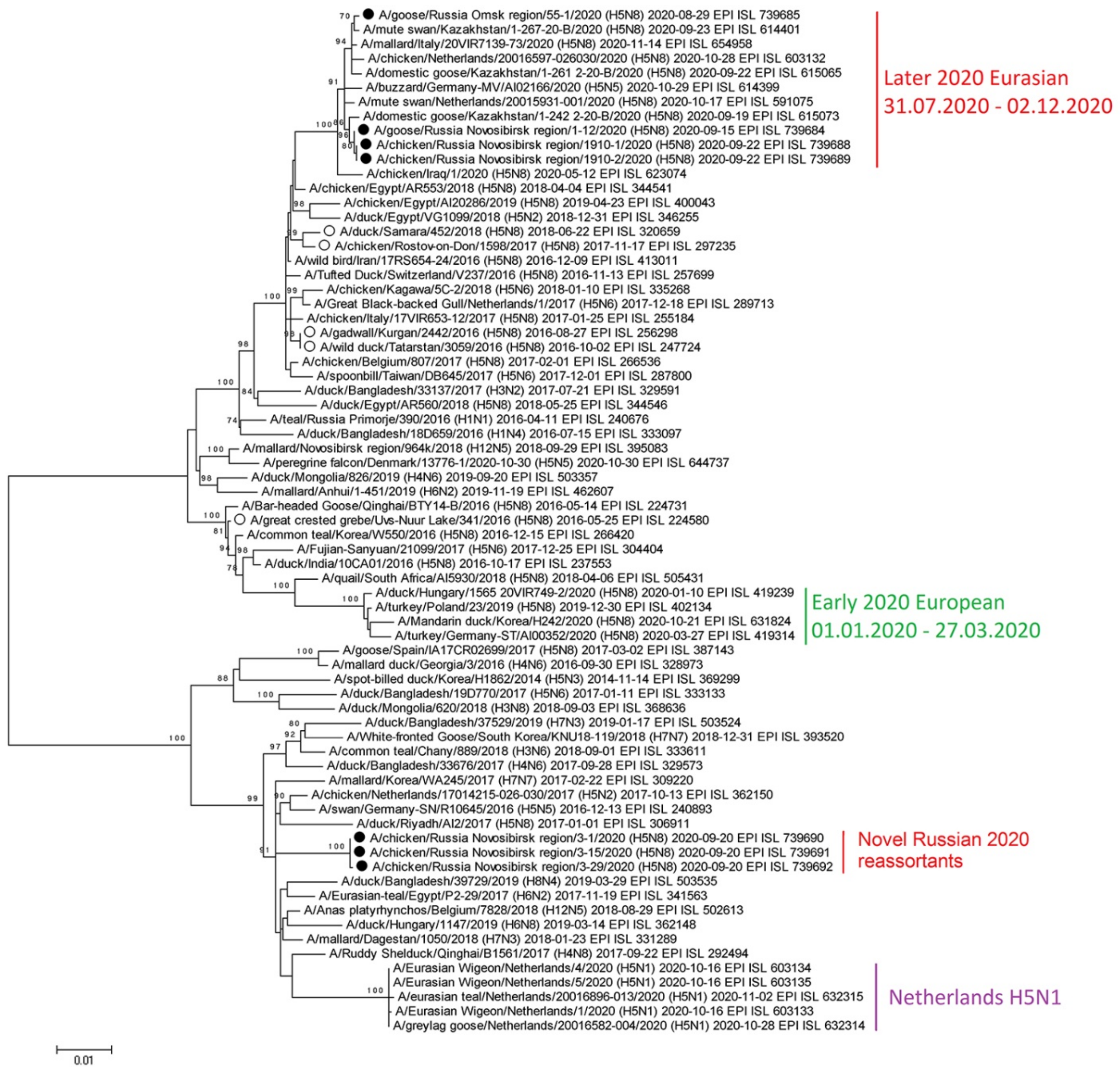

Appendix 1 Figure 2. Maximum-likelihood phylogenetic tree of the PB2 segment. Black circles - HPAIVs H5N8 from Russia isolated in 2020. White circles - Russian HPAIVs H5N8 isolated in 2016-2018.

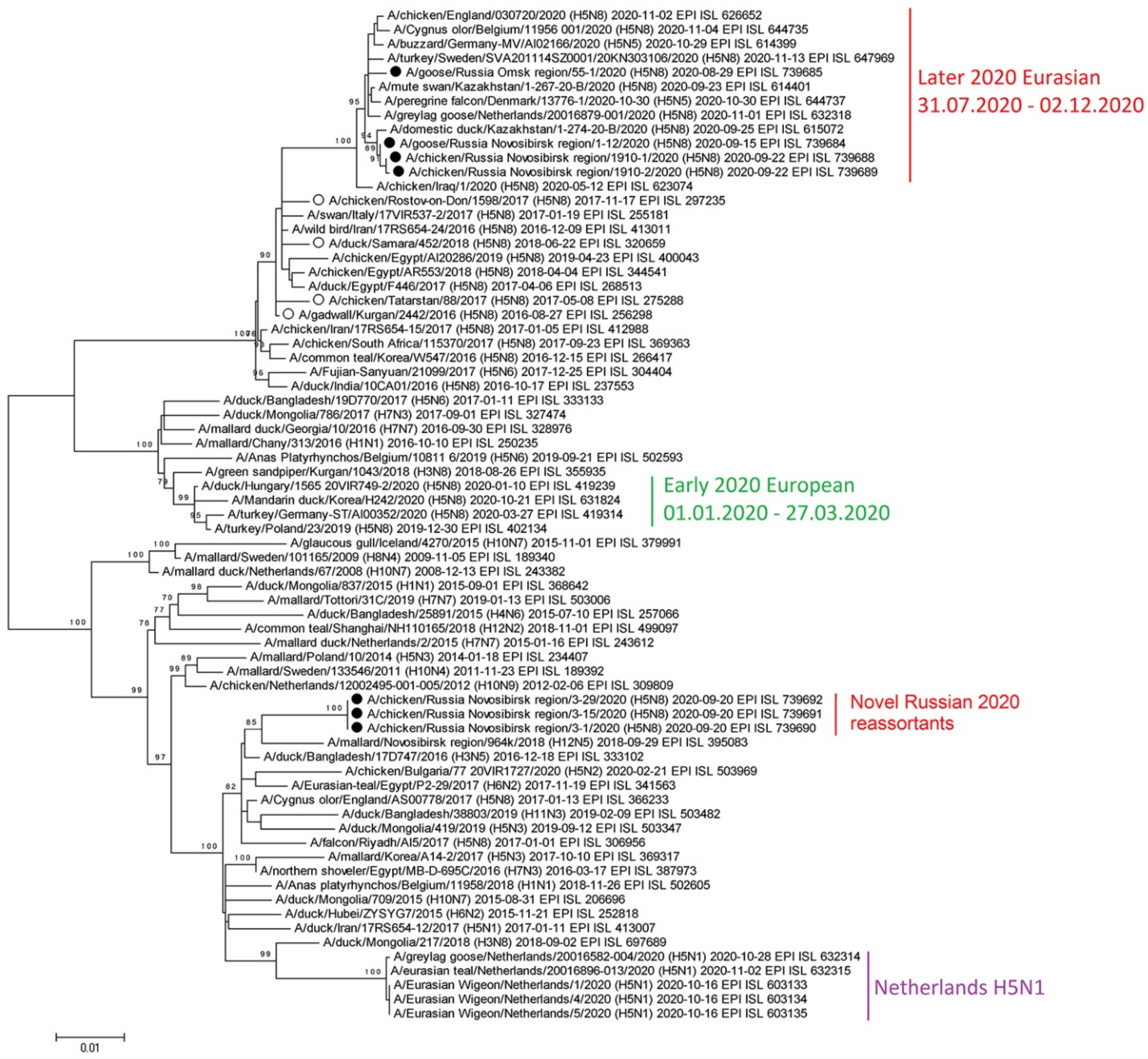

Appendix 1 Figure 3. Maximum-likelihood phylogenetic tree of the PB1 segment. Black circles - HPAIVs H5N8 from Russia isolated in 2020. White circles - Russian HPAIVs H5N8 isolated in 2016-2018.

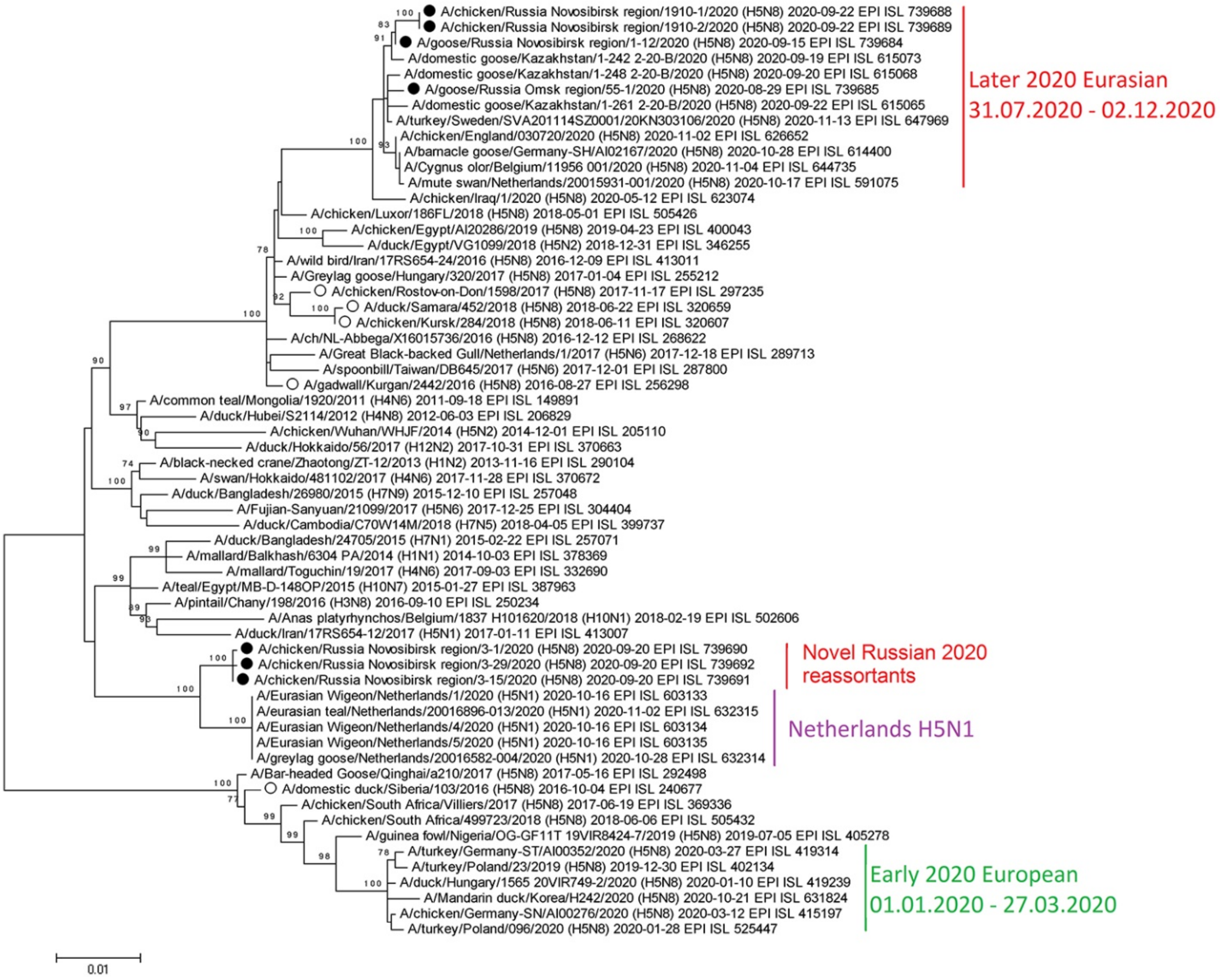

Appendix 1 Figure 4. Maximum-likelihood phylogenetic tree of the PA segment. Black circles - HPAIVs H5N8 from Russia isolated in 2020. White circles - Russian HPAIVs H5N8 isolated in 2016-2018.

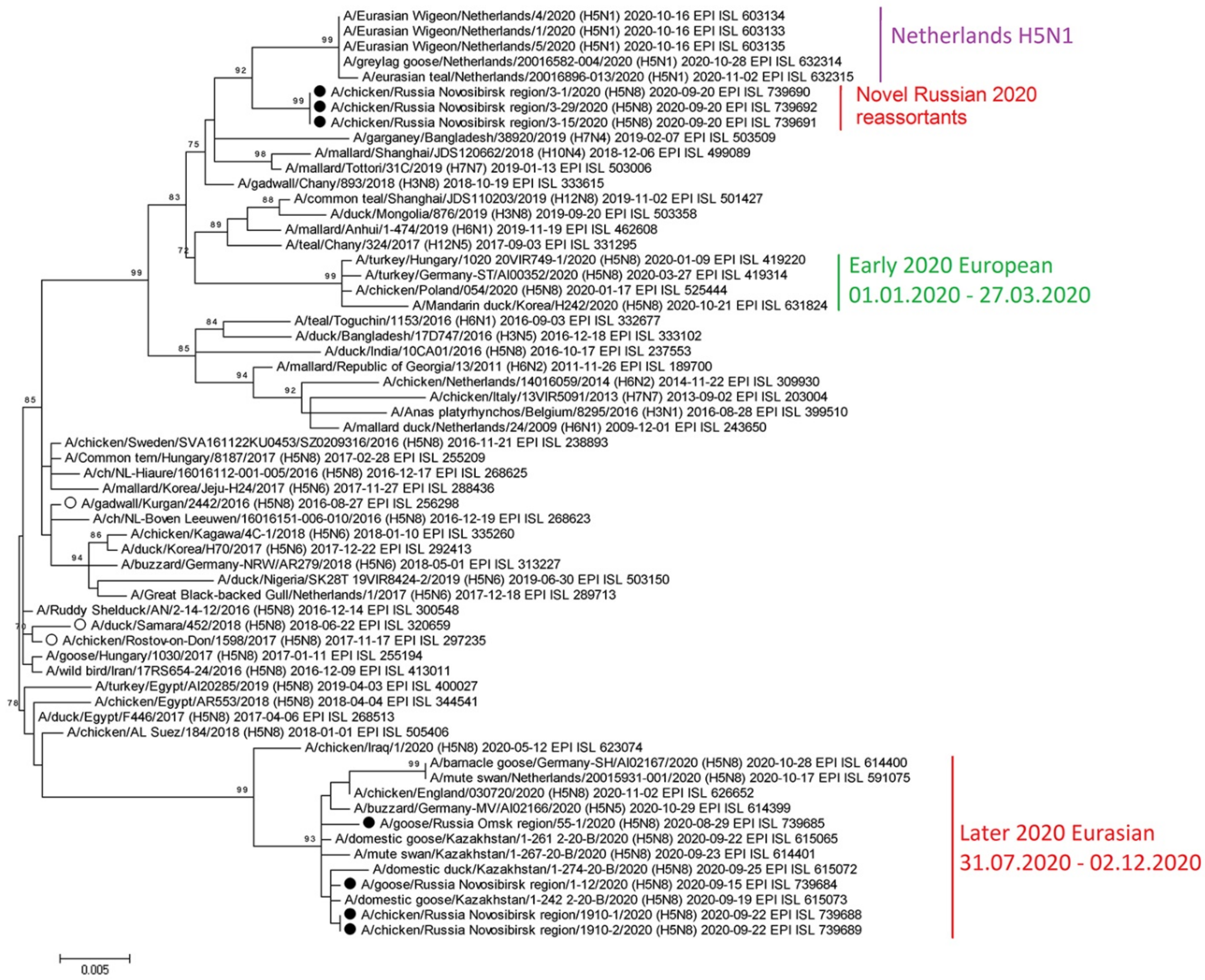

Appendix 1 Figure 5. Maximum-likelihood phylogenetic tree of the NP segment. Black circles - HPAIVs H5N8 from Russia isolated in 2020. White circles - Russian HPAIVs H5N8 isolated in 2016-2018.

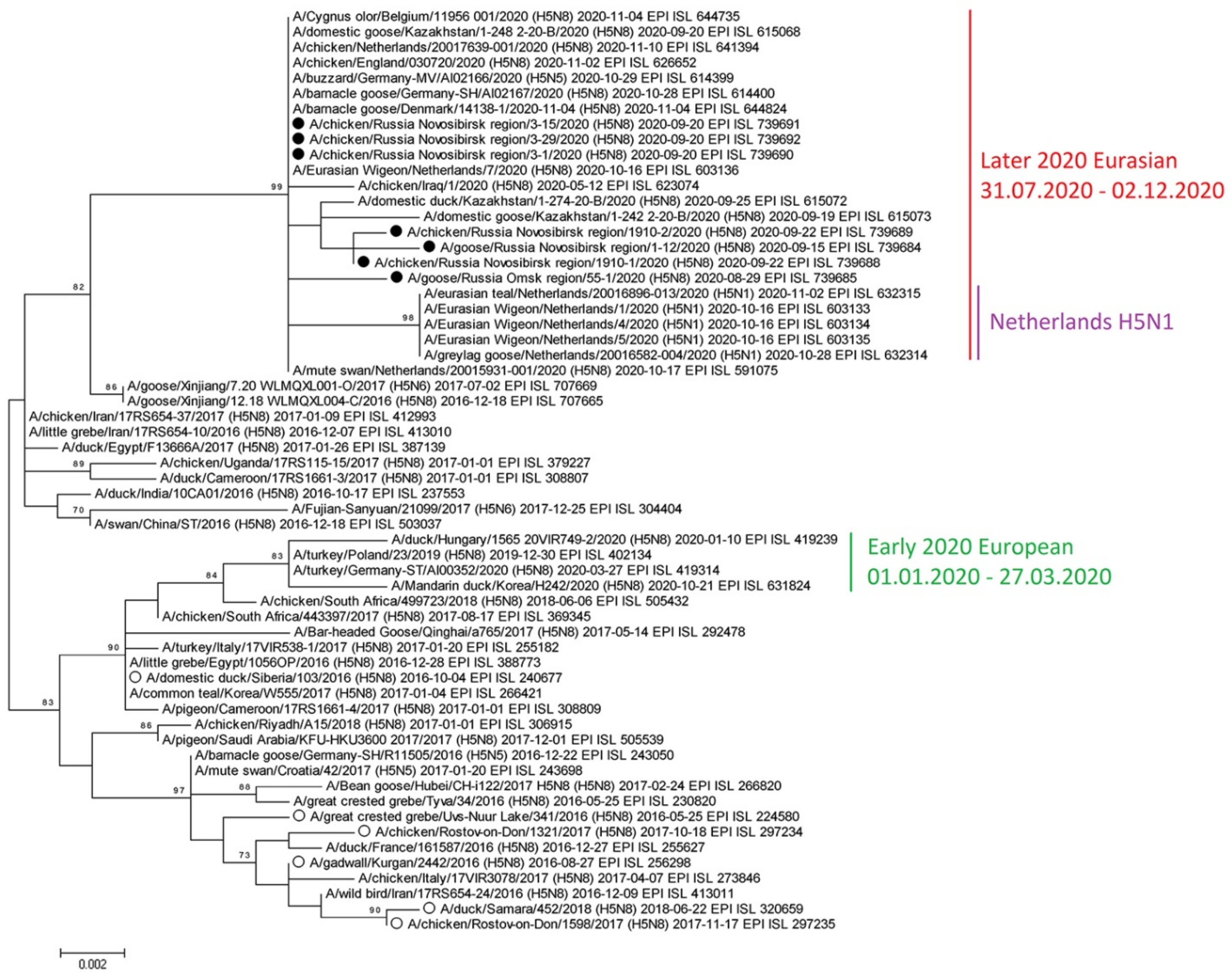

Appendix 1 Figure 6. Maximum-likelihood phylogenetic tree of the MP segment. Black circles - HPAIVs H5N8 from Russia isolated in 2020. White circles - Russian HPAIVs H5N8 isolated in 2016-2018.

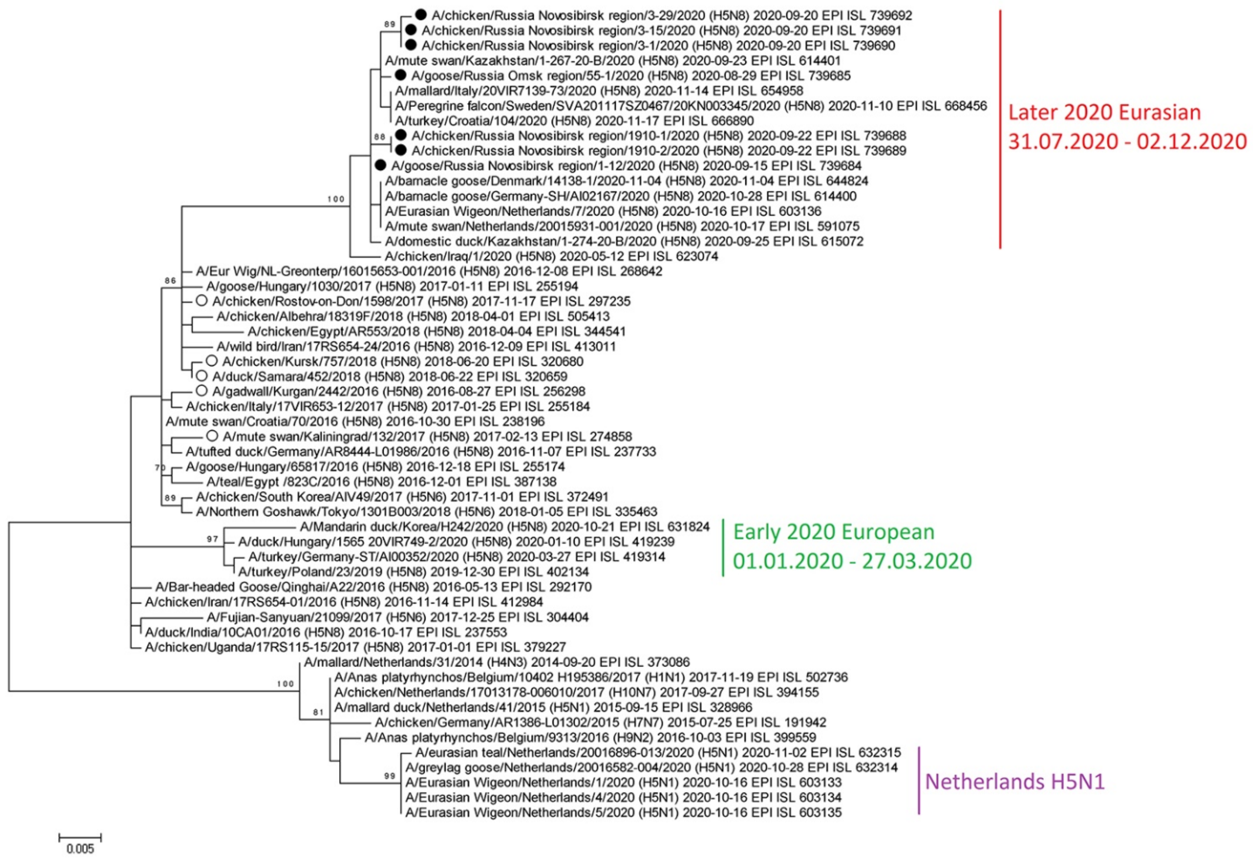

Appendix 1 Figure 7. Maximum-likelihood phylogenetic tree of the NS segment. Black circles - HPAIVs H5N8 from Russia isolated in 2020. White circles - Russian HPAIVs H5N8 isolated in 2016-2018.

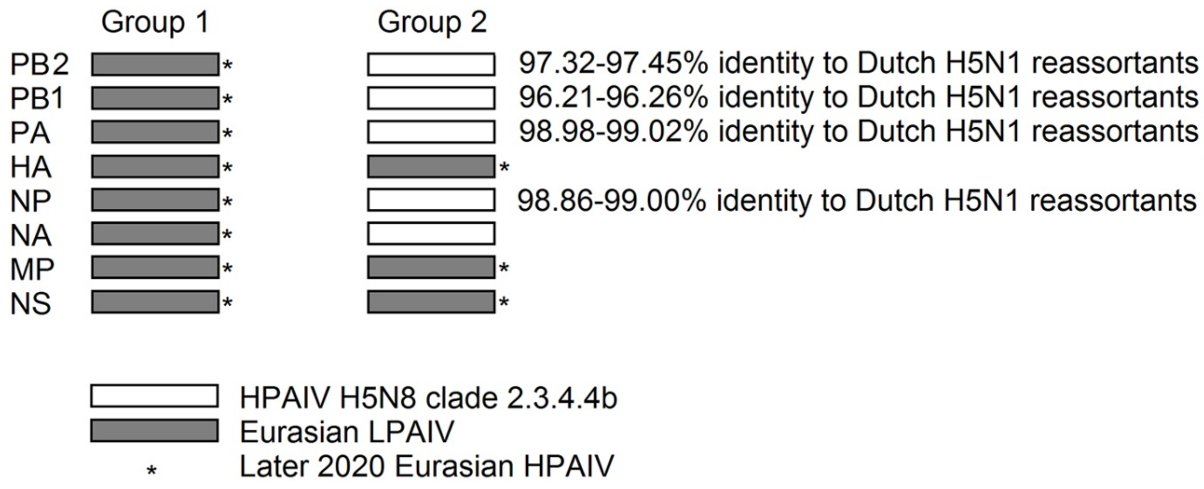

Appendix 1 Figure 8. Differences of the genome segments composition between isolates of groups 1 and 2. The percentage of identity to Dutch H5N1 reassortants is based on matrices of pairwise distances between Russian and Dutch strains (Appendix 1 Figure 10).

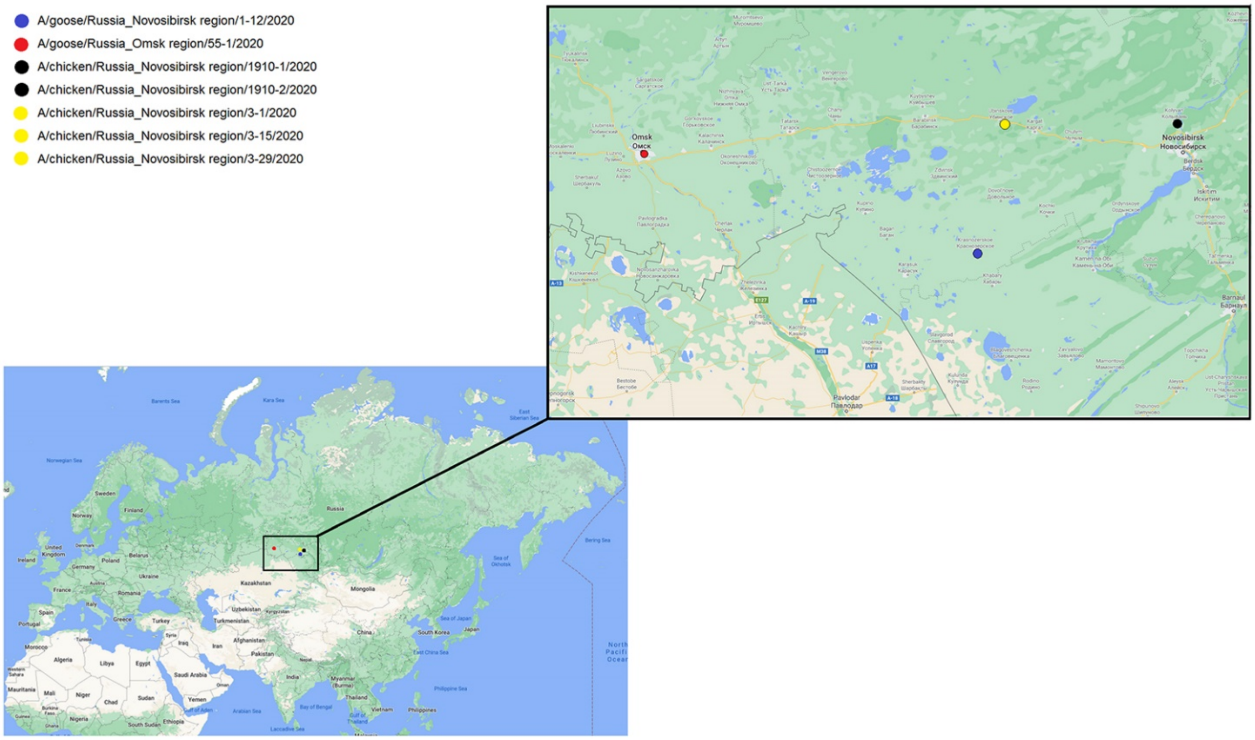

Appendix 1 Figure 9. Sampling locations. Map was retrieved from Google Maps (https://www.google.com/maps).

**PB2**							
A/chicken/Russia_Novosibirsk_region/3-1/2020							
A/chicken/Russia_Novosibirsk_region/3-29/2020	99,96						
A/chicken/Russia_Novosibirsk_region/3-15/2020	100,00	99,96					
A/Eurasian_Wigeon/Netherlands/1/2020	97,36	97,41	97,36				
A/Eurasian_Wigeon/Netherlands/4/2020	97,41	97,45	97,41	99,96			
A/Eurasian_Wigeon/Netherlands/5/2020	97,41	97,45	97,41	99,96	100,00		
A/greylag_goose/Netherlands/20016582-004/2020	97,41	97,45	97,41	99,96	100,00	100,00	
A/eurasian_teal/Netherlands/20016896-013/2020	97,32	97,36	97,32	99,87	99,91	99,91	99,91
**PB1**							
A/chicken/Russia_Novosibirsk_region/3-1/2020							
A/chicken/Russia_Novosibirsk_region/3-29/2020	100,00						
A/chicken/Russia_Novosibirsk_region/3-15/2020	100,00	100,00					
A/Eurasian_Wigeon/Netherlands/1/2020	96,21	96,21	96,21				
A/Eurasian_Wigeon/Netherlands/4/2020	96,21	96,21	96,21	100,00			
A/Eurasian_Wigeon/Netherlands/5/2020	96,21	96,21	96,21	100,00	100,00		
A/greylag_goose/Netherlands/20016582-004/2020	96,21	96,21	96,21	99,91	99,91	99,91	
A/eurasian_teal/Netherlands/20016896-013/2020	96,26	96,26	96,26	99,96	99,96	99,96	99,96
**PA**							
A/chicken/Russia_Novosibirsk_region/3-1/2020							
A/chicken/Russia_Novosibirsk_region/3-29/2020	99,91						
A/chicken/Russia_Novosibirsk_region/3-15/2020	99,95	99,95					
A/Eurasian_Wigeon/Netherlands/1/2020	98,98	98,98	99,02				
A/Eurasian_Wigeon/Netherlands/4/2020	98,98	98,98	99,02	100,00			
A/Eurasian_Wigeon/Netherlands/5/2020	98,98	98,98	99,02	100,00	100,00		
A/greylag_goose/Netherlands/20016582-004/2020	98,98	98,98	99,02	100,00	100,00	100,00	
A/eurasian_teal/Netherlands/20016896-013/2020	98,98	98,98	99,02	100,00	100,00	100,00	100,00
**NP**							
A/chicken/Russia_Novosibirsk_region/3-1/2020							
A/chicken/Russia_Novosibirsk_region/3-15/2020	100,00						
A/chicken/Russia_Novosibirsk_region/3-29/2020	100,00	100,00					
A/Eurasian_Wigeon/Netherlands/1/2020	99,00	99,00	99,00				
A/Eurasian_Wigeon/Netherlands/4/2020	99,00	99,00	99,00	100,00			
A/Eurasian_Wigeon/Netherlands/5/2020	99,00	99,00	99,00	100,00	100,00		
A/greylag_goose/Netherlands/20016582-004/2020	99,00	99,00	99,00	100,00	100,00	100,00	
A/eurasian_teal/Netherlands/20016896-013/2020	98,86	98,86	98,86	99,87	99,87	99,87	99,87

Appendix 1 Figure 10. Matrices of pairwise distances between Russian and Dutch strains.
